# A delayed response in phytohormone signaling and production contributes to pine susceptibility to *Fusarium circinatum*

**DOI:** 10.1186/s12870-024-05342-8

**Published:** 2024-07-30

**Authors:** Laura Hernandez-Escribano, M. Teresa Morales Clemente, David Fariña-Flores, Rosa Raposo

**Affiliations:** 1 Instituto de Ciencias Forestales (ICIFOR-INIA), CSIC, Carretera Coruña km 7.5, Madrid, 28040 Spain; 2 Departamento de Biotecnología-Biología Vegetal, E.T.S. de Ingeniería Agronómica, Alimentaria y de Biosistemas, Universidad Politécnica de Madrid, Madrid, 28040 Spain

**Keywords:** *Pinus radiata*, *Pinus pinaster*, Pine pitch canker, Metabolites, Gene expression, Jasmonic acid, Early defense response, Plant hormones

## Abstract

**Background:**

*Fusarium circinatum* is the causal agent of pine pitch canker disease, which affects *Pinus* species worldwide, causing significant economic and ecological losses. In Spain, two *Pinus* species are most affected by the pathogen; *Pinus radiata* is highly susceptible, while *Pinus pinaster* has shown moderate resistance. In *F. circinatum*-*Pinus* interactions, phytohormones are known to play a crucial role in plant defense. By comparing species with different degrees of susceptibility, we aimed to elucidate the fundamental mechanisms underlying resistance to the pathogen. For this purpose, we used an integrative approach by combining gene expression and metabolomic phytohormone analyses at 5 and 10 days post inoculation.

**Results:**

Gene expression and metabolite phytohormone contents suggested that the moderate resistance of *P. pinaster* to *F. circinatum* is determined by the induction of phytohormone signaling and hormone rearrangement beginning at 5 dpi, when symptoms are still not visible. Jasmonic acid was the hormone that showed the greatest increase by 5 dpi, together with the active gibberellic acid 4 and the cytokinin dehydrozeatin; there was also an increase in abscisic acid and salicylic acid by 10 dpi. In contrast, *P. radiata* hormonal changes were delayed until 10 dpi, when symptoms were already visible; however, this increase was not as high as that in *P. pinaster*. Indeed, in *P. radiata*, no differences in jasmonic acid or salicylic acid production were found. Gene expression analysis supported the hormonal data, since the activation of genes related to phytohormone synthesis was observed earlier in *P. pinaster* than in the susceptible *P. radiata*.

**Conclusions:**

We determine that the moderate resistance of *P. pinaster* to *F. circinatum* is in part a result of early and strong activation of plant phytohormone-based defense responses before symptoms become visible. We suggest that jasmonic acid signaling and production are strongly associated with *F. circinatum* resistance. In contrast, *P. radiata* susceptibility was attributed to a delayed response to the fungus at the moment when symptoms were visible. Our results contribute to a better understanding of the phytohormone-based defense mechanism involved in the *Pinus*-*F. circinatum* interactions and provide insight into the development of new strategies for disease mitigation.

**Supplementary Information:**

The online version contains supplementary material available at 10.1186/s12870-024-05342-8.

## Background

Pine pitch canker (PPC) disease, caused by the fungus *Fusarium circinatum* Nirenberg & O´Donnell, is known to affect more than 60 *Pinus* species [1], causing considerable ecological and economic losses in nurseries and forest plantations worldwide [1–3]. In Europe, the disease was officially reported in Spain in 2005 [4] and thereafter spread throughout its Atlantic coast, representing a serious threat to *P. radiata* and *P. pinaster* plantations. Symptomatic infected mature trees exhibit needle wilting and chlorosis, stem cankers, canopy dieback and mortality, while infected seedlings show wilting and damping-off in nurseries [2, 3]. The susceptibility of different *Pinus* species to the pathogen varies widely [1, 2, 5]. The exotic *P. radiata*, which is highly susceptible to the pathogen [5, 6], is widely planted in northern Spain because of its fast growth and good wood quality [7], contributing positively to the region’s economy. In contrast, *P. pinaster*, which is native to the Mediterranean area and economically significant for resin, timber and paper production, has shown moderate resistance to *F. circinatum* [6]. Despite efforts to find effective management measures, the pathogen has not yet been controlled in nurseries or forest plantations [8, 9].

In recent decades, research on PPC has focused on phenotypic studies in species with different degrees of susceptibility to fungal infection and on isolate pathogenicity [6, 10–15]. However, in recent years, advances in molecular techniques and bioinformatic tools have improved the genetic resources available in forest areas, representing great advances in the knowledge and management of tree diseases [16, 17]. Several transcriptomic studies highlighting the roles of pathogenesis-related (PR) proteins, reactive oxygen species (ROS) production, phytohormone signaling and secondary metabolism (phenylpropanoid pathway and terpenes) related to pine defense against PPC have been published [18–23].

Phytohormones act as signaling molecules that trigger molecular responses to biotic and abiotic stresses [24–26]. These phytohormones also play important roles in regulating plant responses, and cross-talk among different phytohormones is essential for establishing a balanced defenses response [26]. Defense against pathogens is known to be primarily coordinated by jasmonic acid (JA) and ethylene (ET), along with salicylic acid (SA) [27, 28]. This regulatory network has expanded over time to include additional hormones, such as indole acetic acid (IAA), abscisic acid (ABA), cytokinins (Ck) and gibberellic acid (GA), all of which have been implicated in plant defense mechanisms [26]. Regarding PPC, Visser et al. [22] suggested an earlier transcriptomic response based on JA, SA, ET and auxins in the more resistant *P. tecunumanii* and a delayed response in the susceptible *P. patula*. The susceptible *P. radiata* has also shown a weaker induction of phytohormone-related transcripts compared to the resistant *P. pinea*, possibly due to impaired perception of the fungus [21]. The upregulation of ABA signaling-related genes also occurs in *P. radiata* under *F. circinatum* challenge, and its involvement in stomatal opening and closure has been discussed [18]. Davis et al. [29] reported SA and JA induction of chitinases of the PR3 family in *P. elliotti*-resistant genotypes inoculated with *F. circinatum*.

In a previous study [20], we determined the transcriptome profile of *P. pinaster* under *F. circinatum* challenge by a dual RNA sequencing (RNAseq) assay. From this study, we hypothesized that the moderate resistance shown by *P. pinaster* may be explained by recognition of the pathogen from as early as 3 dpi, the induction of pathogenesis-related genes and the activation of complex phytohormone signaling pathways that include mainly SA, JA and ET. We also suggested that *F. circinatum* manipulates the host phytohormone balance to its own benefit through the expression of genes related to hormone biosynthesis. We hypothesized three key steps of host manipulation: perturbing ET signaling via the fungal expression of genes related to ET biosynthesis, blocking JA signaling via coronatine insensitive 1 (*COI1*) suppression, and disturbing SA biosynthesis from the chorismate pathway via the synthesis of isochorismatase family hydrolase (*ICSH*) genes. The blocking of JA signaling by *F. circinatum* has also been proposed by Zamora-Ballesteros et al. [21]. Taken together, these findings suggest the importance of a phytohormone-based defense response against *F. circinatum* infection in *Pinus* species at the transcriptomic level.

In addition to transcriptomics, other ‘*omic* sciences provide a massive amount of information at various molecular levels, including information about DNA, proteins and metabolites. Together, they provide a holistic view of the biological system under study. However, changes such as epigenetic or posttranslational regulatory mechanisms affect different layers of the ‘omic cascade, altering gene expression and ultimately metabolite production [17, 30]. Therefore, only through an ‘*omic*-based integrative approach exploring the variation of genetic resistance of pine species with different degrees of susceptibility to *F. circinatum* we will reveal the biological complexity of the interaction. Metabolomic analysis of the response of *Pinus* to *F. circinatum* revealed that ABA catabolism is a key defense mechanism, with the accumulation of inactive dihydrophaseic acid (DPA) in the needles of susceptible *P. radiata* and weakly ABA-like active phaseic acid (PA) in the resistant *P. pinea* [31]. The ABA concentration increased in both *P. radiata* and *P. pinaster* seedlings upon inoculation [32, 33]. By immunolocalization, ABA and JA accumulation were detected in needles of *P. radiata-* and *P. pinea*-inoculated seedlings, respectively [34]. In addition to hormones, the quantification of other metabolites in response to *F. circinatum* revealed an increase in monoterpenes and diterpenes in *P. radiata*, whereas *P. pinaster* showed no significant changes [35]. Several amino acids accumulate in both *P. radiata* and *P. pinaster* upon inoculation, which is attributed to the induction of secondary metabolite production involved in the defense response [32].

In the present study, we combined gene expression and metabolomic phytohormone analyses to investigate variations in two pine species exhibiting different susceptibility levels to *F. circinatum*: *P. pinaster* (with moderate resistance) and *P. radiata* (highly susceptible). Through an integrated approach encompassing gene expression and metabolomics, we will provide a comprehensive understanding of the genetic and hormonal foundations underlying resistance to *F. circinatum* in these pine species. Based on our findings from a previous transcriptomic study [20], we focused our current research on the activation of the defense response at 5 and 10 days post inoculation (dpi).

## Results

### Differences in susceptibility to *F. circinatum* among *Pinus* species

At the time of sampling (5 dpi), no lesions were observed, while all the seedlings showed lesions at the inoculation site by 10 dpi and needle wilting in *P. radiata* seedlings. Significant differences were detected between species at 21 dpi, with lesion lengths of 3.27 ± 0.31 cm (mean ± standard deviation) and 2.38 ± 0.48 cm for *P. radiata* and *P. pinaster*, respectively (*p* value = 0.024).

### Hormonal response of *P. pinaster* and *P. radiata* to infection and wounding

**Fig. 1:**
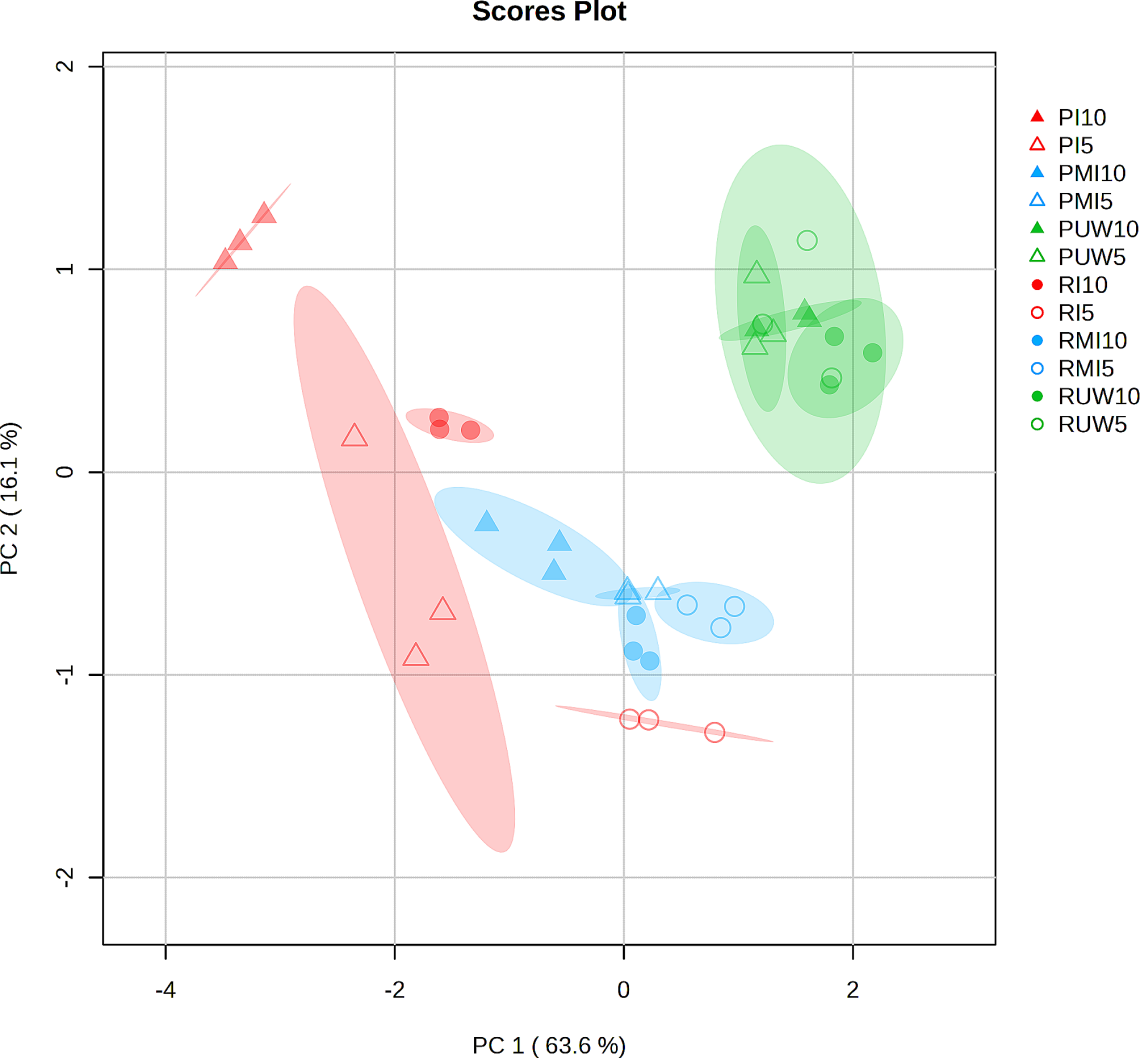
Principal component analysis (PCA) of *P. pinaster* (P) and *P. radiata* (R) seedlings inoculated with *F. circinatum* (I), mock-inoculated (MI) and unwounded (UW) at 5 and 10 days post inoculation. Classes: I5 and I10 for inoculation at 5 and 10 dpi; MI5 and MI10 for mock inoculation at 5 and 10 dpi; and UW5 and UW10 for unwounding at 5 and 10 dpi; P and R for *P. pinaster* and *P. radiata*, respectively

**Fig. 2:**
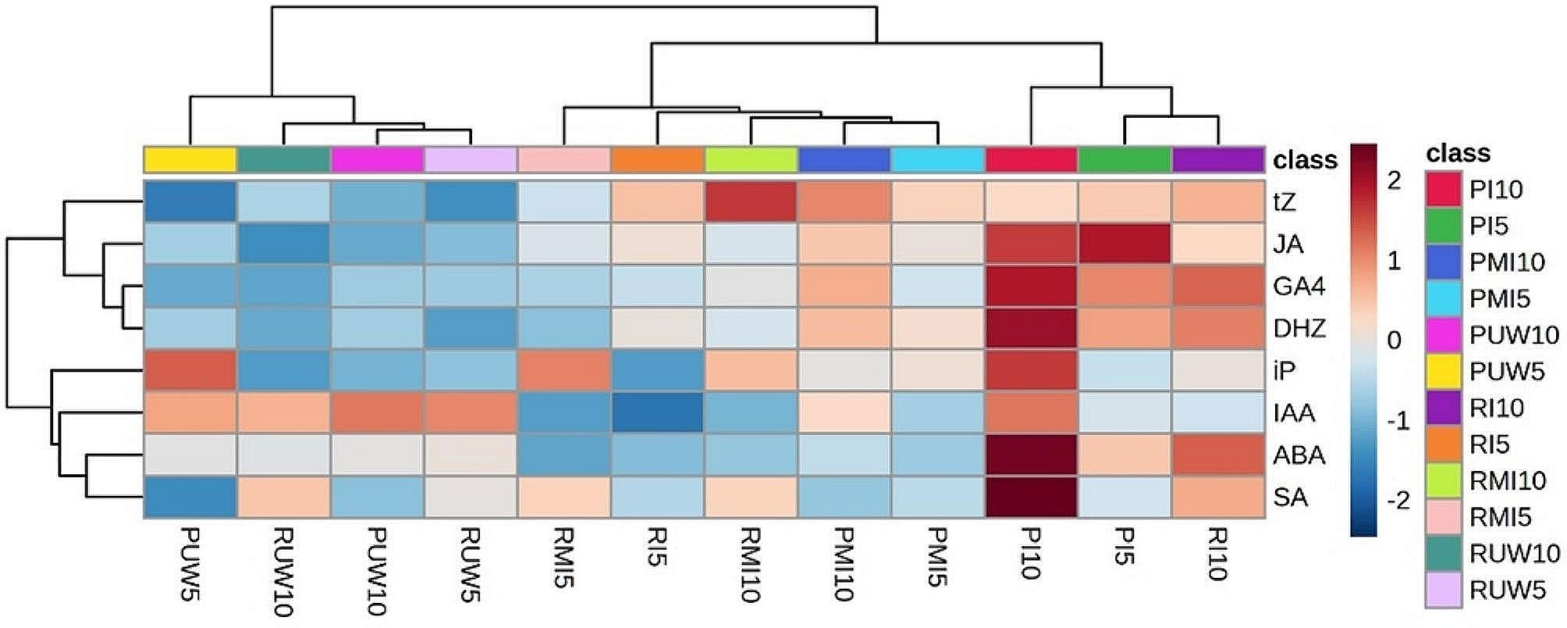
Hormone quantification heatmap of the *P. pinaster* and *P. radiata* datasets. Classes: I5 and I10 for inoculation at 5 and 10 dpi; MI5 and MI10 for mock inoculation at 5 and 10 dpi; and UW5 and UW10 for unwounding at 5 and 10 dpi; P and R for *P. pinaster* and *P. radiata*, respectively. GA_4_: gibberellic acid 4; ABA: abscisic acid; JA: jasmonic acid; IAA: indole acetic acid; SA: salicylic acid; DHZ: dehydrozeatin; tZ: trans-zeatin; iP: isopentenyladenine base

Principal component analysis (PCA), based on the normalized hormone abundances, was performed to determine differences between treatments (Fig. 1). The first two components (PC1 and PC2) explained 79.7% of the total variance, with a major effect associated with PC1 (63.6% of the variance), while PC2 explained 16.1% of the total variance. Separation according to PC1 clustered samples based on their treatment as follows: all unwounded (UW) samples from both pine species were grouped together in one extreme axis, and the other axis was *P. pinaster* inoculated at 10 dpi. Mock-inoculated (MI) seedlings were grouped together and localized close to the inoculated *P. radiata* seedlings at 5 dpi. Hierarchical clustering of hormone content in response to treatments (Fig. 2) classified the seedlings into three clusters: (1) all UW seedlings at 5 and 10 dpi; (2) all MI seedlings, and the seedlings inoculated with *P. radiata* at 5 dpi; and (3) the groups inoculated with *P. pinaster* at 5 and 10 dpi and those inoculated with *P. radiata* at 10 dpi. This plot revealed a delayed response of the hormone abundance of infected *P. radiata*, which clustered at 5 dpi with the MI seedlings, and it was within the group of inoculated *P. pinaster* only at 10 dpi. The highest hormone content was detected in inoculated *P. pinaster* at 10 dpi and for JA at 5 dpi (as indicated by the relatively high red color intensity).

**Fig. 3:**
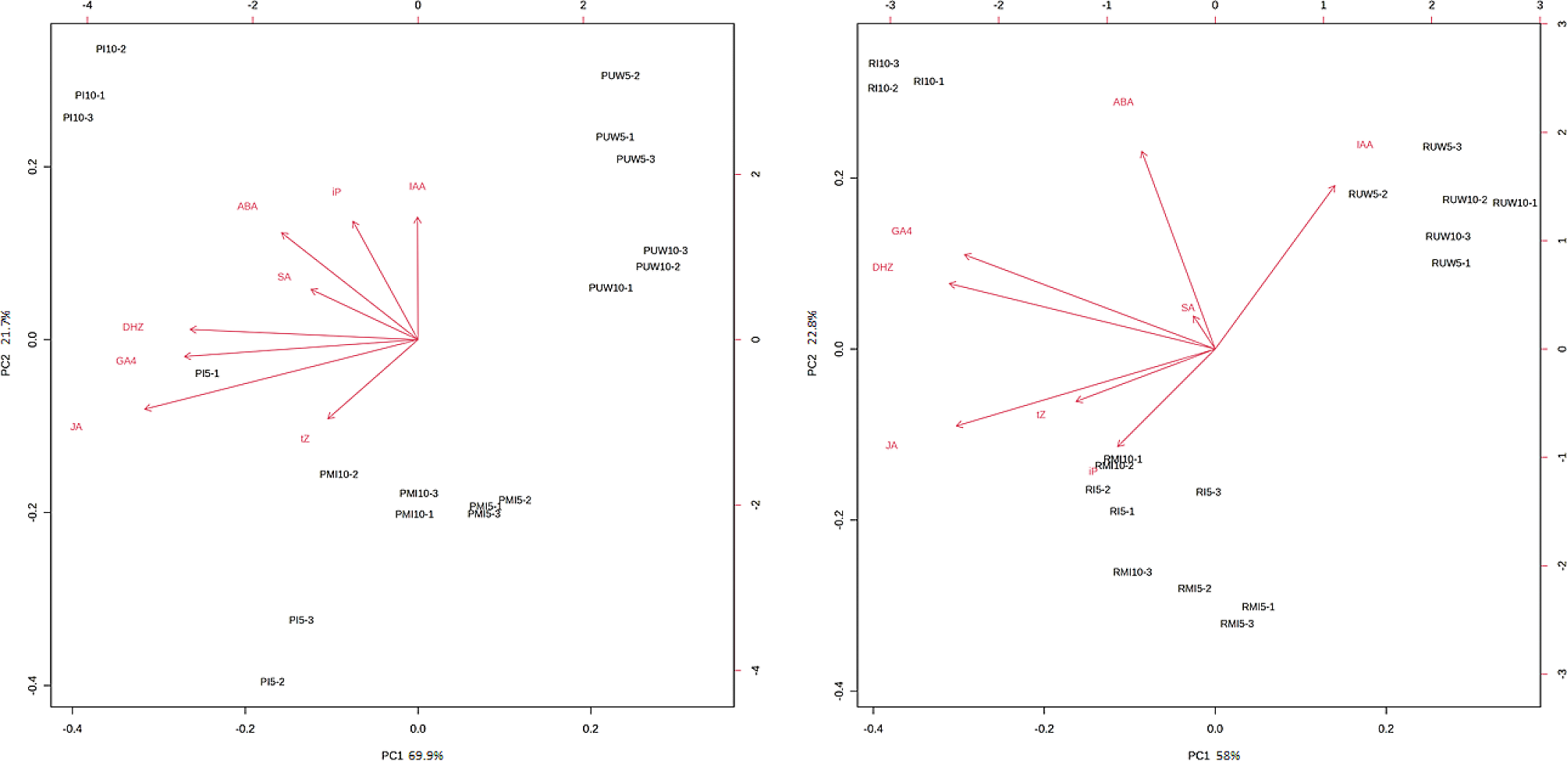
Principal component analysis (PCA) biplots derived from the relative abundances of phytohormones in *P. pinaster* (P) and *P. radiata* (R) seedlings inoculated with *F. circinatum* (I), mock-inoculated (MI) and unwounded (UW) at 5 and 10 days post inoculation. GA_4_: gibberellic acid 4; ABA: abscisic acid; JA: jasmonic acid; IAA: indole acetic acid; SA: salicylic acid; DHZ: dehydrozeatin; tZ: trans-zeatin; iP: isopentenyladenine base. A: *P. pinaster*; B: *P. radiata*

The PCA-based biplot for *P. pinaster* and *P. radiata* highlights the separation of the UW seedlings inoculated at 10 dpi for both *Pinus* species by PC1, representing 70% and 58%, respectively, of the total variance (Fig. 3). Unlike those of *P. radiata*, the inoculated seedlings of *P. pinaster* at 5 dpi did not show a well-formed group, but they were clearly separated from the MI seedlings. GA_4_, dehydrozeatin (DHZ) and JA were the hormones that most influenced the inoculated separation of the *P. pinaster*-inoculated seedlings, as did ABA but to a lesser extent. *P. radiata* inoculation at 10 dpi was strongly correlated with GA_4_, DHZ and JA based on PC1.

**Fig. 4:**
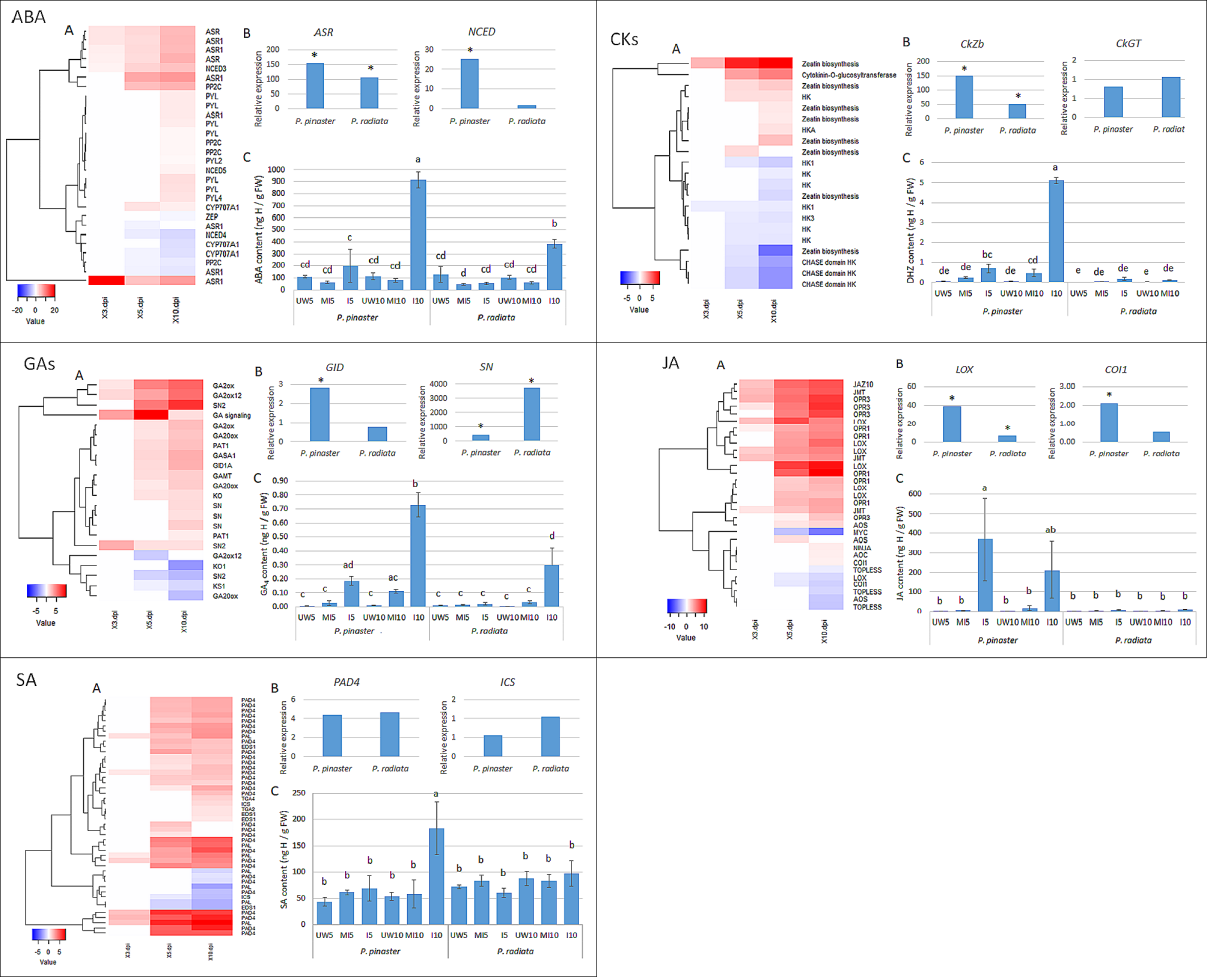
Abscisic acid (ABA), cytokinins (CKs), gibberellic acid (GAs), jasmonic acid (JA), and salicylic acid (SA) production and related gene expression in *Pinus pinaster* and *Pinus radiata* under *Fusarium circinatum* infection. Figure 4A: Heatmap representing differentially expressed genes in *P. pinaster* under *F. circinatum* challenge at 3, 5 and 10 days post inoculation (dpi) as determined by RNA-seq. Figure 4B: The relative expression of candidate genes in *P. pinaster* and *P. radiata* seedlings inoculated with *F. circinatum* at 5 dpi as determined by RT‒qPCR. LOX: lipoxygenase; COI1: coronatine-insensitive protein 1; NCED: 9-cis-epoxy-carotenoid dioxygenase; ASR: abscisic acid-stress-ripening; SN: Snakin/GASA Gibberellin regulated protein; GID: gibberellin receptor; CkGT: cytokinin-O-glucosyltransferase; CkZb: cytokinin hydroxylase; PAD4: lipase; ICS: isochorismatase synthase family. Significant differences (*p* value < 0.05) between inoculated and mock-inoculated seedlings are marked with *. Figure 4C: Phytohormone content (ng of hormone per g of fresh weight) in *P. pinaster* and *P. radiata* seedlings unwounded (UW), mock-inoculated (MI) and inoculated with *F. circinatum* (I) at 5 and 10 dpi. JA, jasmonic acid; ABA, abscisic acid; GA, gibberellic acid; CKs, cytokinins; DHZ, dehydrozeatin; SA, salicylic acid. The different letters above the columns indicate significant differences between treatments (*p* value < 0.05)

**Fig. 5:**
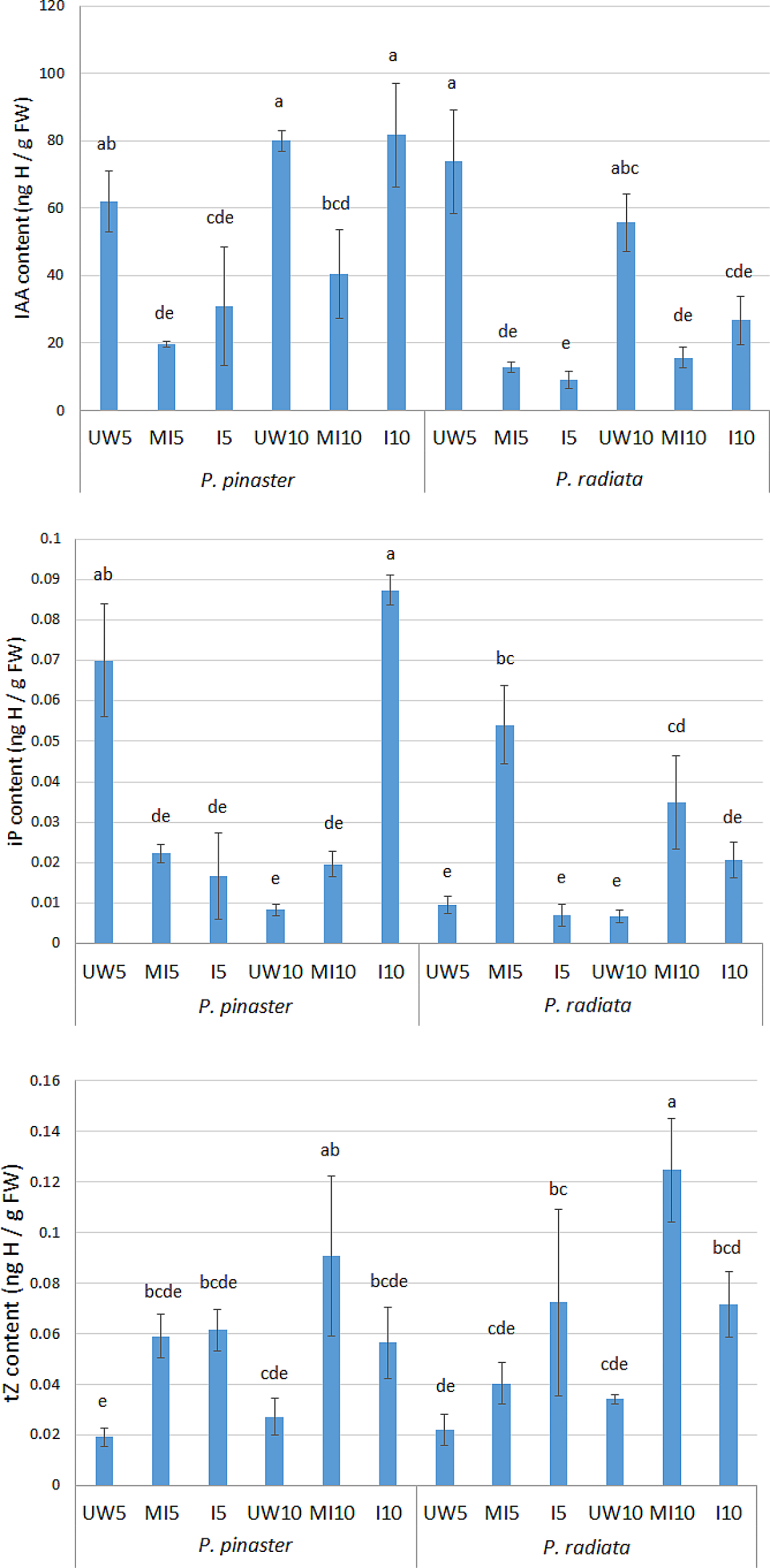
Indole acetic acid (IAA), isopentenyladenine base (iP), and trans-zeatin (tZ) phytohormone contents in *P. pinaster* and *P. radiata* seedlings at 5 and 10 dpi. The data are expressed as ng of hormone per g of fresh weight. The different letters indicate significant differences between samples (*p* value < 0.05)

The effects of *F. circinatum* infection and wounding on *P. pinaster* and *P. radiata* seedlings at 5 and 10 dpi were analyzed with a mixed model (Table 1). The results showed that the effects due to ‘treatment’, ‘species’ and ‘dpi’ were significant for all phytohormones, with the exception of ‘dpi’ for the JA content and ‘species’ for tZ. Pairwise comparisons of the phytohormone contents among species and treatments revealed an earlier response to the pathogen in *P. pinaster* than in *P. radiata*. In *P. pinaster*, the JA, GA_4_ and DHZ metabolite contents increased in response to fungal inoculation as early as 5 dpi, while in *P. radiata*, only GA_4_ and DHZ increased but at 10 dpi (Fig. 4C_JA, GA_4_, Cks). At this time, the GA_4_ and DHZ contents were 2- and 4-fold greater, respectively, in *P. pinaster*. The ABA content increased in both *Pinus* species but increased 2-fold in *P. pinaster* (Fig. 4C_ABA). Notably, the JA content in the inoculated *P. pinaster* at 5 dpi was 65- and 268-fold greater than that in the MI and UW treatments, respectively (Fig. 4C_JA). The SA content increased in the *P. pinaster*-inoculated samples by 10 dpi (3-fold greater than the MI and UW seedlings), while no differences were found in the SA content of *P. radiata* (Fig. 4C_SA). None of these phytohormones showed any significant change in response to wounding (no significant differences were observed between the UW and MI treatments). However, a significant effect of wounding was observed for indole acetic acid (IAA), isopentenyladenine base (iP) and trans-zeatin (tZ) (Fig. 5). Both pine species reduced the IAA content in response to wounding at 5 and 10 dpi, while tZ increased in the MI seedlings at 10 dpi. iP increased at 5 and 10 dpi in *P. radiata* MI seedlings but only at 5 dpi in *P. pinaster*.

**Table 1:** Type 3 tests for fixed effects in a mixed model including species, treatment and days postinoculation

	GA_4_	ABA	IAA	JA	SA	DHZ	iP	tZ
Sp	**59.46**	**50.38**	**36.79**	**15.56**	0.15	**299.4**	**41.8**	2.31
Treat	**160.56**	**147.39**	**65.45**	**15.69**	**11.73**	**501.08**	**6.74**	**31.85**
dpi	**107.43**	**112.87**	**21.2**	0.98	**17.63**	**364.63**	0.02	**14.71**
Sp*Treat	**38.41**	**43.99**	**8.24**	**14.31**	**13.12**	**203.71**	**68.4**	0.18
Sp*dpi	**14.68**	**17.17**	**19.16**	1.03	2.82	**151.7**	1.08	3.22
Treat*dpi	**75.56**	**107.55**	**9.14**	1.18	**12.01**	**306.14**	**88.41**	**10.69**
Sp*Treat*dpi	**6.77**	**13.71**	0.7	1.27	**3.91**	**133.56**	**51.76**	2.04

Species: Sp; treatment: Treat; days pos tinoculation: dpi. GA4: gibberellic acid 4; ABA: abscisic acid; JA: jasmonic acid; IAA: indole acetic acid; SA: salicylic acid; DHZ: dehydrozeatin; tZ: trans-zeatin; iP: isopentenyladenine base. Values in bold are significant (*p* value < 0.05)

**Fig. 6:**
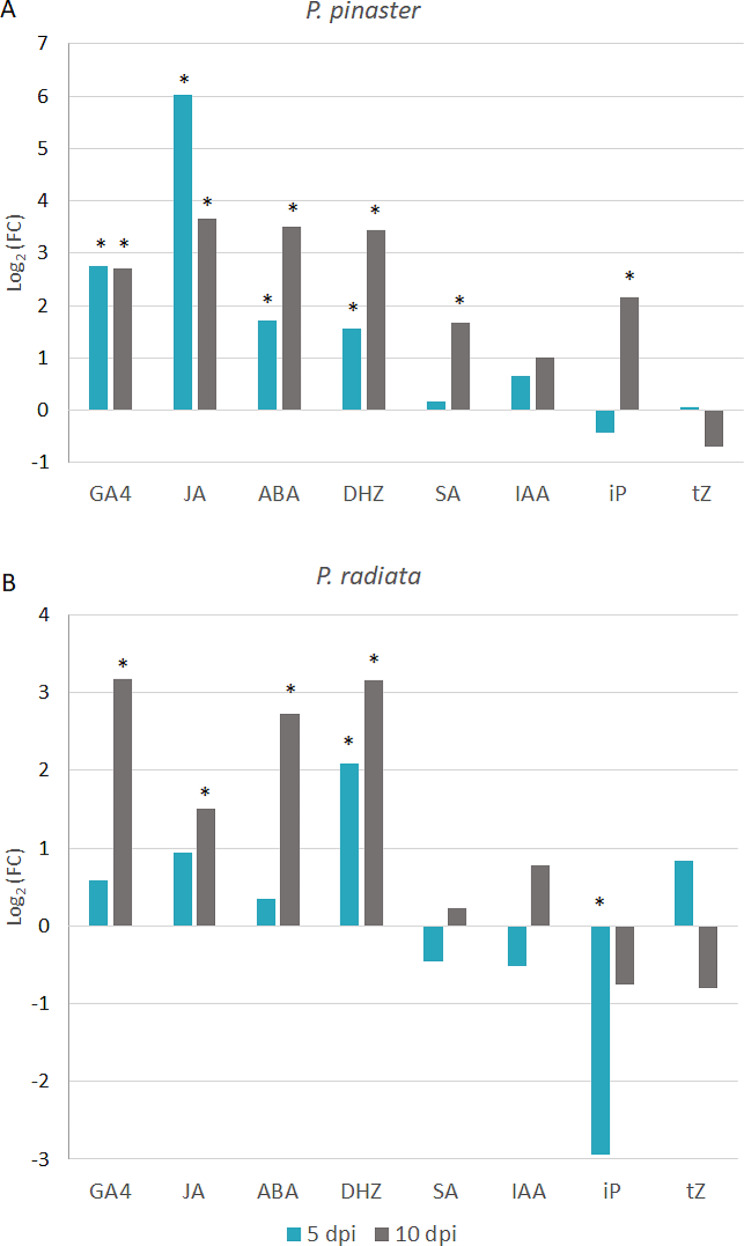
Hormone content (represented as Log_2_FC) in infected seedlings relative to mock-inoculated seedlings of *P. pinaster* (**A**) and *P. radiata* (**B**) at 5 and 10 dpi. An asterisk (*) indicates a fold change (FC) greater than |1.5| that is also statistically significant (*p* value < 0.05).value GA_4_: gibberellic acid 4; ABA: abscisic acid; JA: jasmonic acid; IAA: indole acetic acid; SA: salicylic acid; DHZ: dehydrozeatin; tZ: trans-zeatin; iP: isopentenyladenine base. A: *P. pinaster*; B: *P. radiata*

A further comparison between the inoculated and MI treatments at each dpi for each *Pinus* species highlighted the quick activation of the *P. pinaster* phytohormone-based response to *F. circinatum*. The log_2−_fold change (FC) values of GA_4_, ABA, DHZ and JA were significantly greater in the inoculated *P. pinaster* seedlings compared to MI from as early as 5 dpi (Fig. 6A). Notably, JA significantly increased by 5 dpi, with a log_2_(FC) value of 6 (Fig. 6A). Among these phytohormones, only DHZ increased in *P. radiata* at this time point, with a value similar to that of *P. pinaster* (Fig. 6B). At 10 dpi, DHZ, JA, GA_4_ and ABA increased due to fungal infection in both *Pinus* species, with higher values in *P. pinaster*, except for GA_4_ (Fig. 6A and B). Compared with those in the MI seedlings, the SA and iP contents in the inoculated *P. pinaster* samples increased by 10 dpi (Fig. 6A).

The constitutive hormone content refers to the hormone content in UW seedlings, and both *Pinus* species had similar compositions. ABA, IAA and SA were the most abundant, followed by JA, with Ck (DHZ, iP and tZ) and GA_4_ being less abundant (Figs. 4C and 5). Significant differences were found only for iP in *P. pinaster*, with a greater difference at 5 dpi (Fig. 5).

### Updating *P. pinaster* transcriptomic hormone response to *F. circinatum*

**Fig. 7:**
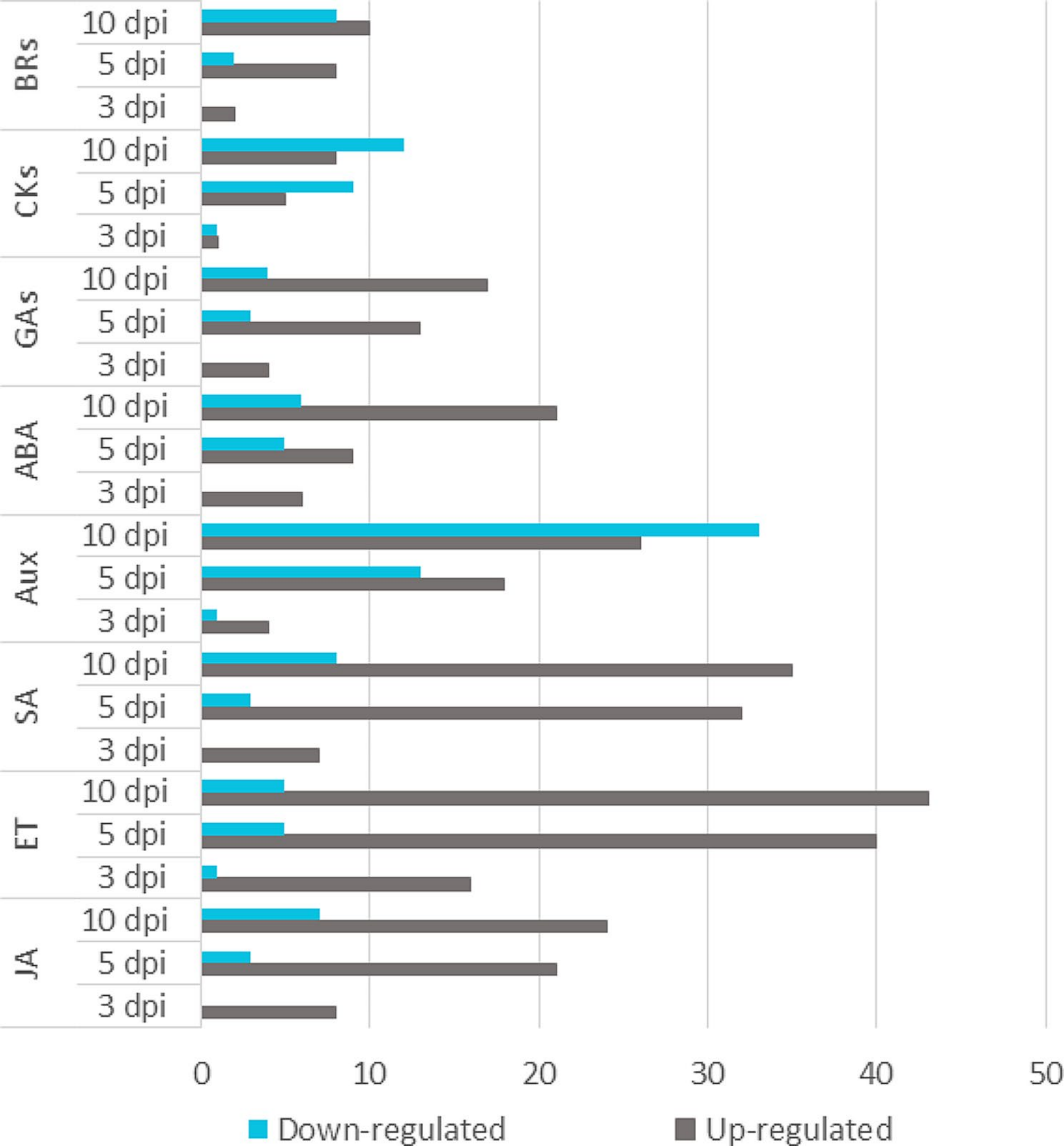
Total number of differentially expressed genes (DEGs) from dual RNA sequencing analysis of *P. pinaster* under *F. circinatum* infection at 3, 5 and 10 days post inoculation (dpi) (updated from Hernandez-Escribano et al. [20]). Up- or downregulated genes were determined by comparing the expression levels in inoculated samples with those in the mock-inoculated samples at each dpi. BR: brassinosteroid; CKs: cytokinins; GA: gibberellin; ABA: abscisic acid; Aux: auxins; SA: salicylic acid; ET: ethylene; JA: jasmonic acid

We revisited the transcriptome dataset of *P. pinaster* under *F. circinatum* infection at 3, 5 and 10 dpi determined previously using a dual RNAseq assay [20]. In this work, we reported that JA, ET and SA play major roles in the *P. pinaster* defense response. A deeper study focused on hormones revealed new differentially expressed genes (DEGs) related to GA, ABA and Ck phytohormones. A total of 45 new DEGs were identified and added to the set of 234 DEGs initially identified (Fig. 7, Additional Table 1). With respect to ABA signaling, eight *ASR* genes (abscisic stress ripening proteins) and seven abscisic acid receptor genes were DEGs and were mostly upregulated at 5 and 10 dpi. Several GA-regulated proteins (*GASA*, *SN2*) and a GA receptor (*GID1*), as well as several *GA20 oxidase* genes, were also upregulated at 5 and 10 dpi. Within Cks, some zeatin biosynthesis-related genes were DEGs and were up- and downregulated at 5 and 10 dpi. These newly identified DEGs suggest that not only JA, SA, and ethylene but also ABA and GA are key players in the *P. pinaster* transcriptomic response to *F. circinatum* (Fig. 4A).

### Relative expression of genes related to phytohormones by RT‒qPCR

Two candidate genes for each of the quantified hormones (JA, ABA, GA, Cks and SA) were selected for relative expression analysis in *P. pinaster* and *P. radiata*. The expression of these genes differed between inoculated and MI samples at 5 dpi according to the pine species considered (Fig. 4B). In *P. pinaster*, *F. circinatum* infection led to an increase in the abundance of transcripts related to JA (*LOX*, *COI1*), ABA (*ASR*, *NCED*), GA4 (*GID*, *SN*) and DHZ (*CkZb*) signaling. In *P. radiata*, only the relative expression of *LOX*, *ASR*, *SN* and *CkZb* increased due to fungal infection. For both pine species, *SN* was the gene with the greatest fold change, at 417-fold for *P. pinaster*, while its expression in *P. radiata* was observed only in inoculated samples (with almost no expression in MI samples) (Fig. 4B_GAs). The relative expression of all other genes was greater in *P. pinaster*. No significant differences were found in the relative expression of SA-related genes at 5 dpi in either of the pine species (Fig. 4B_SA).

### Stomatal conductance of needles

**Fig. 8:**
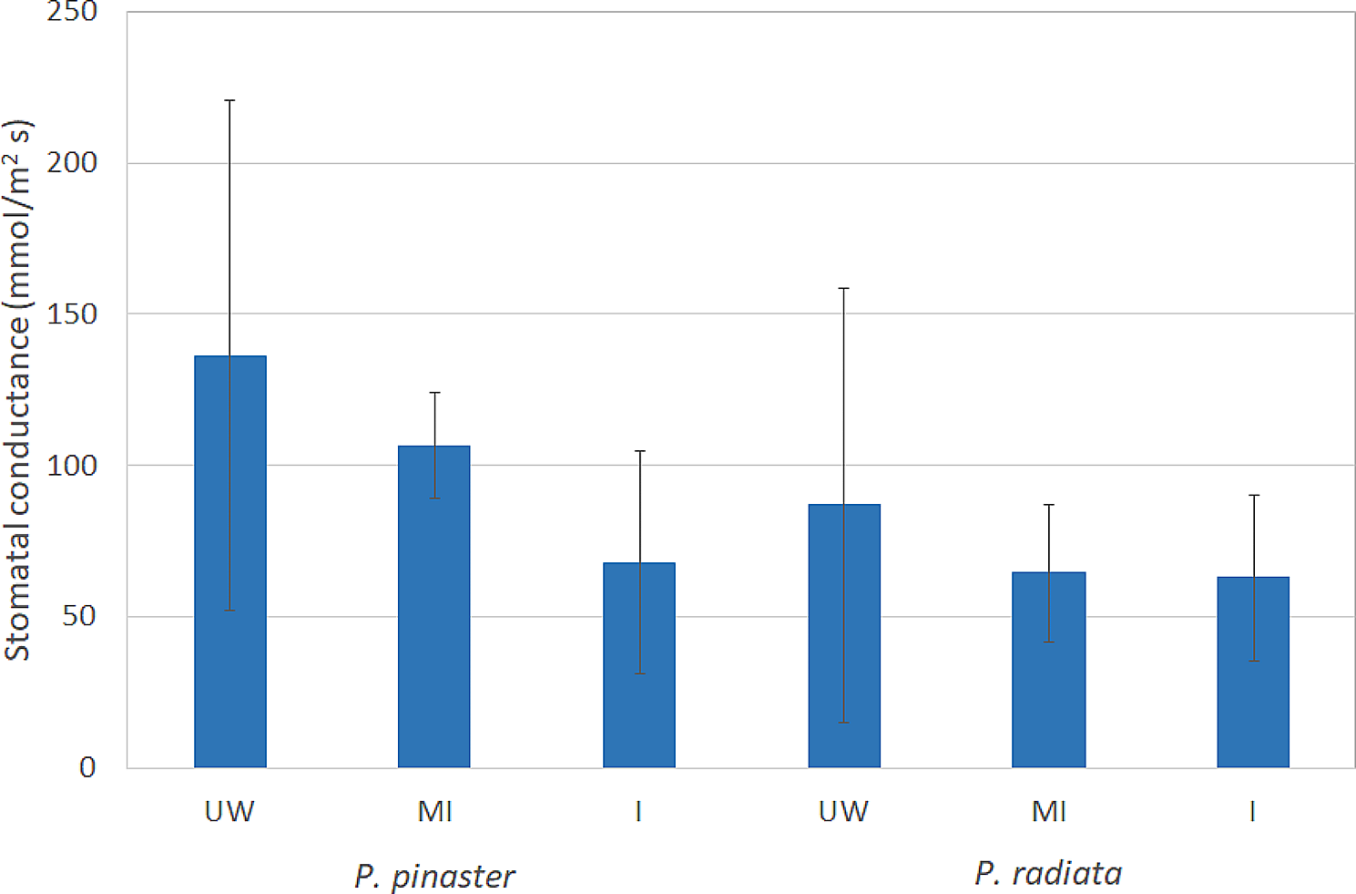
Stomatal conductance (expressed in mmol/m^2^ s) of *P. pinaster* and *P. radiata* needles at 10 days after treatment. UW: unwounded; MI: mock-inoculated; I: inoculated with *F. circinatum*

*Fusarium circinatum* did not significantly alter the stomatal conductance of the pine seedlings (Fig. 8). Nevertheless, both the inoculated and mock-inoculated *P. pinaster* and *P. radiata* seedlings exhibited reduced stomatal conductance compared to the unwounded treatment, but were not significant at the 0.05 statistical level.

## Discussion

In this work, we studied the hormone content in *P. pinaster* and *P. radiata* as a defense response to *F. circinatum*. The combination of gene expression and hormonal analyses indicated that the moderate resistance of *P. pinaster* to *F. circinatum* is associated with the prompt activation of a phytohormone-based defense response, based mainly on JA. This phenomenon becomes evident as early as 5 dpi, preceding the onset of initial symptoms. In contrast, the susceptibility exhibited by *P. radiata* could be attributed at least in part to a delayed response to fungal infection, mainly at the moment when symptoms are visible (10 dpi). The phytohormone abundance data based on the clustering analysis and PCA support this hypothesis since the *P. radiata*-inoculated samples at 5 dpi clustered together with the MI samples, suggesting that the response to wounding was greater than that to the pathogen at this time point. Indeed, the heatmap color intensity of the *P. pinaster*-inoculated group indicated that a substantial increase in hormone content had already occurred in response to *F. circinatum* at 5 dpi and increased from 5 to 10 dpi.

JA is often associated with defense against herbivores and necrotrophic pathogens and usually acts in synergy with ET [36]. *Fusarium circinatum* is a hemibiotrophic fungus with a necrotrophic phase when infecting *Pinus* seedlings [37]. Interestingly, the increase in JA in inoculated *P. pinaster* seedlings at 5 dpi and the slight increase in the susceptible *P. radiata* seedlings by 10 dpi (Fig. 6) is in agreement with previous results. The JA concentration increased in the needles of *P. pinaster* seedlings infected with *F. circinatum* by 17 dpi [32], the time at which at least 50% of the inoculated seedlings display symptoms, while no changes were observed in the inoculated *P. radiata* seedlings by 10 dpi [32]. However, our results revealed an earlier increase in JA in the stems of inoculated *P. pinaster* seedlings by 5 dpi, before symptoms became visible (Fig. 4C_JA). In a recent study, using the hormone immunolocalization technique, the JA signal was shown to increase rapidly in the needles of resistant *P. pinea* under *F. circinatum* infection from 2 h post inoculation to 10 dpi and was maintained during the time course [34]. JA accumulation as a plant response to other pathogens has also been reported, as in needles and roots of *P. sylvestris* under infection by the pathogen *Diplodia pinea* [38].

The transcriptomic data also supported the involvement of JA in the moderate resistance of *P. pinaster* to PPC. We previously reported the upregulation of several genes related to JA biosynthesis (*LOX*, *OPR*, *AOC*, *AOS*) and signaling (*COI1*, *TPL*, *NINJA*, *JMT*) from the early stage at 3 dpi [20]. In the present study, the RT‒qPCR results showed an increase in *COI1* and *LOX* transcripts in *P. pinaster* seedlings at 5 dpi (Fig. 4B_JA). *COI1* is the receptor that perceives the active form of JA, jasmonoyl-isoleucine (JA-Ile), and *LOX* is a lipoxygenase enzyme that catalyzes the essential steps in JA biosynthesis [39, 40]. In contrast, *COI1* expression did not change in the susceptible *P. radiata* at 5 dpi, suggesting that no signaling occurred at this time point. Similarly, Zamora-Ballesteros et al. [21] determined that the number of genes involved in JA signaling was considerably greater in resistant *P. pinea* than in susceptible *P. radiata* during *F. circinatum* infection. Taken together, these findings emphasize the importance of JA in the response of *P. pinaster* to PPC beginning in the early stages of infection, even before the first symptoms become visible. We propose that JA is a key hormone in the early defense response of *P. pinaster* against *F. circinatum* that contributes to pine resistance.

In contrast to JA, SA is commonly involved in the response to biotrophic or hemibiotrophic fungi [36, 41–43]. The antagonistic effects of JA and SA have been largely reported [41, 44–46], but crosstalk between these two phytohormones and cooperation rather than antagonism have also been reported [47–49]. Previous studies did not report any changes in the SA content in *P. pinea*, *P. pinaster* or *P. radiata* under *F. circinatum* infection at 64, 17 and 10 dpi, respectively, when 50% of the seedlings displayed symptoms [32, 34]. Our findings do not support these results, possibly because of factors such as the sampling time or seedling age. We showed that the SA content increased at 10 dpi in *P. pinaster* due to pathogen infection, which was the time at which we detected the first symptoms at the inoculation site (Fig. 4C_SA). By RT‒qPCR, we detected no differences in the relative expression of the two genes related to SA at 5 dpi in any of the pine species (Fig. 4B_SA). However, in a deeper study based on transcriptomic data analysis, we found several DE genes related to SA biosynthesis and signaling in *P. pinaster*, mainly at 5 and 10 dpi [20]. This could explain the accumulation of SA we detected in *P. pinaster* at 10 dpi. We previously reported the upregulation of the isochorismatase family hydrolase (*ICSH*) gene in *F. circinatum* following *P. pinaster* infection, and proposed that *F. circinatum* could manipulate SA signaling through the chorismatase pathway [20]. In this context, the susceptibility of *P. radiata* may be due in part to its inability to overcome this imbalance. However, whether *F. circinatum* manipulates host hormone homeostasis requires further studies using knockout mutants. Taken together, these findings collectively indicate that SA likely plays a role in the defense response of *P. pinaster* but not in the initial stage.

The role of ABA as a positive [50] or negative [51, 52] regulator of plant defense has been reported. ABA accumulation in *P. pinaster* and *P. radiata* was detected when symptoms were visible upon *F. circinatum* infection [32], which is consistent with the results presented here. Furthermore, we found that the increase in ABA content at 10 dpi was more pronounced in *P. pinaster* than in *P. radiata* (Fig. 4C_ABA), and the ABA content in inoculated *P. radiata* seedlings did not increase significantly until 10 dpi compared to the MI seedlings, contributing to an earlier response in *P. pinaster* compared to *P. radiata* (Fig. 6). RNA-seq analysis [20] suggested that ABA signaling does not seem to play a major role in the *P. pinaster* response to *F. circinatum*. However, reinspection of the data revealed new upregulated transcripts related to ABA biosynthesis and signaling (Additional Table 1; Fig. 7). Similarly, ABA signaling-related genes were DEGs in *P. radiata* and *P. pinea* upon *F. circinatum* inoculation [18, 21]. The results of the RT‒qPCR analysis at 5 dpi revealed the induction of both the *ASR* and *NCED* genes in the inoculated *P. pinaster* seedlings, while only *ASR* was induced in the *P. radiata* seedlings, and the relative expression of these genes was decreased (Fig. 4B_ABA). These results suggest a stronger ABA-mediated response in *P. pinaster* than in *P. radiata*.

A delayed hormone response in *P. radiata* compared to *P. pinaster* under *F. circinatum* challenge was also detected for GA_4_ (Fig. 4C_GAs). In the metabolomics analysis of plant-pathogen interactions, the difficulty of discriminating between plant and pathogen metabolites poses a particular challenge [53]. Indeed, some fungi-produced hormones, such as ABA, GA and ET, are known to contribute to pathogenicity [54]. *Fusarium circinatum* belongs to the *F. fujikuroi* species complex and is known to produce GA, resulting in host hormone imbalance and contributing to plant disease [55, 56]. Unlike metabolites, transcripts depend on a known genetic sequence and allow the identification of the organism of origin [57]. We identified genes related to GA biosynthesis and signaling via *in silico* analysis of *P. pinaster* and *F. circinatum* transcriptomes during the infection process, suggesting that GA is produced not only by the plant but also by the fungus [20]. By RT‒qPCR at 5 dpi, we found that the relative expression of both *SN* (Snakin Gibberellin regulated protein) and *GID* (Gibberellin receptor) increased in the moderately resistant *P. pinaster*, while the expression of only *SN* was increased in *P. radiata*. Remarkably, the relative expression of *SN* in both *Pinus* species increased in response to the fungus at this time point. Therefore, gene expression analysis related to GA supports the hormonal data and the delayed response observed in *P. radiata*. Whether a portion of the quantified metabolite GA_4_ originates from fungal metabolite production needs further investigation.

Cks are adenine derivatives with isoprenoid side chains that can be categorized as isopentenyl adenine (iP), trans-zeatin (tZ), cis-zeatin (cZ) or dihydrozeatin (DHZ)-type derivatives depending on the hydroxylation of the isoprenoid side chains. Cks regulate numerous processes involved in plant growth and development [58, 59], including plant responses to abiotic and biotic stresses [60]. Among the three zeatin-type Cks (DHZ, tZ and iP) quantified in this study, the DHZ content increased due to fungal infection at both time points in *P. pinaster* but only at 10 dpi in *P. radiata*, indicating a delay in the hormonal response (Fig. 5). The difference in the iP content was not significant until 10 dpi and was greater in *P. pinaster* (Fig. 5). By RT‒qPCR, we found that the relative expression of *CkZb* increased in both *Pinus* species at 5 dpi (Fig. 4C_CKs). Although the role of Cks in plant defense is not clear, crosstalk with other phytohormones, such as SA and JA, has been proposed [60–62]. For example, Cks are needed for JA-mediated resistance of *Arabidopsis* to the necrotroph *Alternaria alternata* [63]. We observed an effect of wounding on Ck production: the iP content increased in *P. radiata* MI seedlings but decreased in *P. pinaster* seedlings at 5 dpi (Fig. 5), and the tZ content increased in response to wounding in both *Pinus* species at 10 dpi (Fig. 5). We also observed an inhibitory effect of wounding on IAA production in both *Pinus* species beginning at 5 dpi (Fig. 5). Regarding infection, the results showed that IAA does not play a major role in the response to *F. circinatum*.

Physiological changes regulated by hormonal metabolism have also been investigated in *Pinus*-*F. circinatum* interactions. Dieback and girdling cankers in branches with symptoms of PPC are known to occur as a result of water flow obstruction after *F. circinatum* infection [10, 64]. Under pathogen infection, plants are known to control transpirational water loss by regulating stomatal opening and closure, which is mainly controlled by ABA signaling [65]. Stomatal closure is also mediated by JA [66–68], and a role for Ck in preinvasive defense via the induction of stomatal closure has been documented [69]. Stomatal opening and increased transpiration rates in the highly resistant *P. pinea* upon inoculation with *F. circinatum* were reported [32], while stomatal closure and photosynthesis impairment were found in *P. radiata* and *P. pinaster* [32, 33, 70]. Although our data suggest a decrease in stomatal conductance in inoculated and MI *P. pinaster* and *P. radiata* seedlings by 10 dpi, no significant differences were found. Similarly, Zamora-Ballesteros et al. [21] reported that stomatal conductance was not influenced by the pathogen in *P. radiata* and *P. pinea* seedlings, although differences were found between species, with stomatal conductance being greater in *P. pinea*. The connection of ABA accumulation with stomatal conductance and its correlation with PPC disease has been a subject of discussion [34, 70].

The constitutive content of the studied phytohormones did not determine the outcome of the interaction, as indicated by the fact that no differences were found between *P. pinaster* and *P. radiata* in the unwounded seedlings, neither wounded or inoculated with *F. circinatum*. This suggests that the induction of these phytohormones is more closely related to fungal resistance/susceptibility than to the basal hormone content.

## Methods

### Plant material and fungal culture

Eight-month-old *P. pinaster* and *P. radiata* seedlings were used for the experiment and were obtained from Valladolid (Castilla y León, Spain) and Basque Country (Northern Spain) nurseries, respectively. Seedlings were maintained at 22 °C under a 14/10 h light/dark photoperiod and inoculated after one month of acclimation. Plants were irrigated regularly when needed.

Pine seedlings were inoculated with a virulent strain of *F. circinatum* isolated from an infected *P. radiata* tree located in Basque Country (isolate CECT20759, [13]). The fungus was grown on potato dextrose agar (PDA, Condalab, Madrid, Spain) plates at 22 °C in the dark for 6 days. A spore suspension was prepared by using a hemocytometer and adjusted to a final concentration of 5 × 10^5^ spores/mL with sterile distilled water.

### Inoculation and tissue sampling

*Pinus pinaster* and *P. radiata* seedlings were distributed into three groups: those inoculated with *F. circinatum*, those mock-inoculated with sterile distilled water (MI), and those neither wounded nor inoculated (UW). For inoculation, the first two cm of the shoot tip of each seedling in the inoculated or MI group was excised [20]. For inoculated seedlings, a 2 µl drop of the spore suspension was deposited in the wound, while sterile distilled water was used for the MI seedlings. Plants were covered with plastic bags during the first 24 h after inoculation to promote fungal infection.

Tissues were collected at 5 and 10 dpi. We selected these time points based on a previous transcriptomic assay in which the greatest changes in the expression of phytohormone-related genes were detected [20]. The experiment consisted of six different groups for each *Pinus* species: those inoculated at 5 and at 10 dpi, those mock-inoculated at 5 and at 10 dpi and those unwounded at 5 and at 10 dpi; the groups were named I5 and I10, MI5 and MI10, and UW5 and UW10, respectively. We used three biological replicates for each class, and each biological replicate consisted of a pool of eight seedlings. The top 1.5 cm of shoot tissue, from which the needles were removed, was harvested and immediately frozen in liquid nitrogen. For the UW seedlings, the first 2 cm was removed. The tissue was ground into powder with a mortar and pestle and maintained at -80 °C until use.

A set of five seedlings for each species was maintained for 21 days for visual observation to assess disease symptoms. At this time point, the lesion length was measured, and the shoot tips of all the seedlings were cultured in PDA media to verify the efficacy of the inoculation.

### Plant hormone quantification in *P. pinaster* and *P. radiata*

Hormone quantification of *P. pinaster* and *P. radiata* samples was performed at the Institute for Plant Molecular and Cellular Biology (IBMCP-UPV-CSIC, Valencia, Spain) using a metabolomics platform. Eight hormones were quantified: jasmonic acid (JA), salicylic acid (SA), active gibberellic acid 4 (GA_4_), abscisic acid (ABA), indole acetic acid (IAA), the active cytokinins dehydrozeatin (DHZ), trans-zeatin (tZ) and isopentenyladenine base (iP).

Ground tissue (approximately 50–200 mg fresh weight) was suspended in 80% methanol/1% acetic acid containing internal standards and mixed by shaking for one hour at 4 °C. The extract was kept at -20 °C overnight and then centrifuged, and the supernatant was dried in a vacuum evaporator. The dry residue was dissolved in 1% acetic acid and passed through a reversed-phase column (HLB Oasis 30 mg, Waters) [71]). The extracts were additionally passed through an Oasis MCX (cation exchange). For GA_4_, IAA, ABA, SA and JA quantification, the dried eluates were eluted with 100% methanol/1% acetic acid to recover the acid fraction. In cases where the resolution of the peaks was poor, the extracts were further purified by passing through an Oasis WAX (ion exchange) column eluted with 80% methanol/1% acetic acid. Cytokinins (Cks) were eluted with 60% methanol/5% NH_4_OH from the Oasis MCX column to obtain the basic fraction containing Cks.

The final residues were dried and dissolved in 5% acetonitrile/1% acetic acid, and the hormones were separated by UHPLC with a reverse Accucore C18 column (2.6 μm, 100 mm length; Thermo Fisher Scientific) with an acetonitrile gradient containing 0.05% acetic acid at 400 µL/min for the acid hormones. For GA_4_, IAA, ABA, SA and JA, the gradient was 2 to 55% acetonitrile over 21 min. For Cks, the acetonitrile gradient was 2 to 25% over 13 min.

The hormones were analyzed with a Q-Exactive mass spectrometer (Orbitrap detector; Thermo Fisher Scientific) by targeted selected ion monitoring (tSIM; capillary temperature 300 °C, S-lens RF level 70, resolution 70.000) and electrospray ionization (spray voltage 3.0 kV, heater temperature 150 °C, sheath gas flow rate 40 µL/min, auxiliary gas flow rate 10 µL/min) in negative mode for acidic hormones or positive mode for Cks.

The concentrations of hormones in the extracts were determined using embedded calibration curves and the Xcalibur 4.0 and TraceFinder 4.1 SP1 programs. The internal standards for the quantification of each of the different plant hormones were deuterium-labeled hormones, except for JA, for which the compound dhJA was used (purchased from OlChemim Ltd., Olomouc, Czech Republic). For each sample, the final metabolite content was expressed as ng of hormone per g of fresh weight tissue.

### Relative gene expression by RT?qPCR in *P. pinaster* and *P. radiata*

To characterize the DEGs in *P. pinaster* and *P. radiata*, ten candidate hormone-related genes were selected on the basis of the *P. pinaster* transcriptome under *F. circinatum* infection ( [20]; BioProject accession number PRJNA543723). The selected transcript IDs are found in Additional Table 1. Total RNA from inoculated and MI seedlings at 5 dpi was extracted using a Plant/Fungi Total RNA Purification Kit (Norgen Biotek Corp., Thorold, Ontario) and treated with a TURBO DNA-free kit (Thermo Fisher Scientific) following manufacturer’s instructions. The integrity and concentration of the RNA were measured using a Nanodrop (NanoDrop 2000, Thermo Fisher Scientific, MA, USA), and the RNA was stored at -80 °C until further use. cDNA was synthesized from 1 µg of total RNA using an iScript cDNA synthesis kit (Bio-Rad, Barcelona, Spain) following the manufacturer’s instructions.

Primer3Plus v.3.3.0 software [72] was used to design specific primers. Actin (ACT) [19] and ubiquitin (UBQ) [73] were selected as reference genes for normalizing the relative expression profiles of *P. radiata* and *P. pinaster*, respectively. The efficiency of all primers was checked, and primers with an efficiency less than 90% were discarded. The reactions were run on a StepOnePlus Real-Time PCR System (Applied Biosystems, CA, USA) with the SYBR Green (NZYSpeedy qPCR Green Master Mix 2x; NZYTech, Lisbon, Portugal) detection method. A 1:10 dilution of 2 µl of cDNA was added to 10 µl of SYBR Green mix. The PCR program consisted of an initial denaturation step (2 min at 97 °C) followed by 40 cycles of denaturation (5 s at 95 °C) and annealing (30 s at 60 °C). After amplification, a melting step was performed at 95 °C for 15 s, 60 °C for 1 min, and 95 °C for 15 s. Three technical replicates of each of the three biological replicates were used. The FC of the relative gene expression between the inoculated and MIMI seedlings was calculated by the 2^–∆∆Ct^ method [74].

### Stomatal conductance of needles

The stomatal conductance (expressed in mmol/m^2^ s) of the needles was measured with a leaf porometer (Leaf Porometer Model SC-1, Decagon Devices) at 10 dpi. For each pine species, three seedlings for each treatment (UW, inoculated or MI) were measured. Each replicate consisted of three measurements of the needles of the apex. The back side of a pool of 5 needles was measured each time to cover the measurement surface of the porometer.

### Statistical analysis

A mixed model was used to analyze the effect of ‘species’ (*P. radiata, P. pinaster*), ‘days post inoculation’ (5 dpi, 10 dpi) and ‘treatment’ (inoculated, MI or UW) on the total hormone content. All factors were considered fixed, and the model included two- and three-order interaction factors. Pairwise comparisons of the least square means for all effects were performed using Tukey’s post hoc test at a significance level of 0.05. Analysis was performed with SAS Studio 3.8 (SAS Institute Inc., Cary, NC, USA) (Table 1).

Metaboanalyst 5.0 software (https://www.metaboanalyst.ca) was used for the metabolome analysis. The data was previously normalized to adjust for systematic differences via logarithmic base 10 transformation and Pareto scaling [75]. The data were subjected to PCA to reveal the structure of the samples. A heatmap clustering analysis was conducted using each metabolite abundance based on the Euclidean distance and performed using the Ward clustering method. To better discriminate which phytohormones are produced in response to *F. circinatum*, the log_2_ FC of each phytohormone between the inoculated and MI groups was calculated at each dpi for each *Pinus* species. For this purpose, we set a log_2_ FC threshold of |1.5| and a significance level of 0.05.

For the RT‒qPCR data, Student’s’ t test was performed to estimate the significance difference between the normalized Ct values of the inoculated and MI samples for each gene in each pine species (*p* ≤ 0.05). Student’s t test was also used to determine significant differences in the final lesion length between the *P. pinaster-* and *P. radiata*-inoculated seedlings.

For the stomatal conductance data analysis, a mixed model was fitted that included ‘species’ (*P. radiata, P. pinaster*) and ‘treatment’ (inoculated, MI or UW) as fixed factors with two-factor interaction terms. Pairwise comparisons of the least square mean for all effects were performed using Tukey’s test at a significance level of 0.05.

## Conclusions

We conclude that moderate *P. pinaster* resistance is, at least in part, a result of rapid and strong activation of phytohormone-based defense responses. In contrast, we propose that *P. radiata* susceptibility to *F. circinatum* is explained by a delayed response in gene activation associated with phytohormone biosynthesis and signaling, which ultimately leads to a postponed production of hormone metabolites. The selection of pine species exhibiting different susceptibility levels to PPC enables a comparative analysis of the molecular mechanisms associated with resistance and susceptibility. This valuable information can contribute to the identification of key factors involved in resistance, enhancing our understanding of *Pinus*-*F. circinatum* interactions. Such insights are crucial for developing effective and innovative disease control measures, thereby aiding in disease mitigation strategies related to PPC.

### Electronic supplementary material

Below is the link to the electronic supplementary material.

Supplementary Material 1

Supplementary Material 2

Supplementary Material 3

## Data Availability

Raw sequence data were accessed at NCBI BioProject accession PRJNA543723.

## Abbreviations

SA: salicylic acid, GAs: gibberellins, ET: ethylene
JA: jasmonic acid
CKs: cytokinins
IAA: indole acetic acid
ABA: abscisic acid
DHZ: dehydrozeatin
GA4: gibberellic acid 4
tZ: trans-zeatin
iP: isopentenyladenine base, dpi: days postinoculation
PPC: pine pitch cankerMI: mock-inoculated
UW: unwounded
PCA: principal component analysis
FC: fold change
DEG: differentially expressed genes
PC: principal component
LOX: lipoxygenase
COI1: coronatine-insensitive protein 1
NCDE: 9-cis-epoxy-carotenoid dioxygenase
ASR: abscisic acid-stress-ripening
SN: snakin/GASA
GID: gibberellin receptor
CkGT: Cytokinin-O-glucosyltransferase
CkZb: cytonikin hydroxylase
PAS4: lipase
ICS: isochorismatase synthase family
ACT: activin
UBQ: ubiquitin
PR: pathogenesis-related
ICSH: isochorismatase family hydrolase
DPA: dihydrophaseic acid: PA: phaseic acid
