# Supplementary material for: A delayed response in phytohormone signaling and production contributes to pine susceptibility to Fusarium circinatum
Source: BMC Plant Biol. 2024 Jul 30;24:727. doi: 10.1186/s12870-024-05342-8 (PMC11289988; doi:10.1186/s12870-024-05342-8)
Supplement: Supplementary file 2 — Supplementary Material 2 [file 12870_2024_5342_MOESM2_ESM.docx]

Additional Table 2: Primer sequences of candidate genes related to phytohormone signaling used in RT-qPCR assay.

| Related hormone | Primer ID | Primer sequence  Forward/ Reverse | Description | Transcript ID  (BioProject PRJNA543723) |
| --- | --- | --- | --- | --- |
| JA | LOX | CTCAACGATTTTAACGCAGGCA  TCACTGACAAGCTCGAACGA | Lipoxygenase (JA biosynthesis) | A05_TRINITY_DN21748_c0_g1_i1 |
| JA | COI1 | TCTCTCAGGTGTGCAAGCAG  CTTCAGTGACTCCAACCGCT | Coronative-insensitive protein 1 (JA signaling) | B03_TRINITY_DN45298_c1_g1_i4 |
| ABA | NCED | CGACGATCCTCTCAGAAGCC  GAAACTTTGGGCCACGGTTC | 9-cis-epoxy-carotenoid dioxygenase (ABA biosynthesis) | C08_R4009912 |
| ABA | ASR | CCAGCTCATCTCATCGGCAA  CGTCTTTCCTTGCCCTCTCA | Abscisic acid-stress-ripening (ASR) (ABA signaling) | B04_TRINITY_DN61698_c1_g3_i1 |
| GAs | SN | TGTGGGGTACGATGCCAAAA  GGAGGAACACAACTGCACCT | Snakin/GASA (Gibberellin regulated protein) | C04_R10666440 |
| GAs | GID | GATTCTGCATCAAACCGGCC  CAAAACACAGCACGGCGTTA | GID1 (Gibberellin receptor) | C10_J3548926 |
| Cks | CkGT | CGAGCAGCCGACTAACAGAA  AGATCCCCCTTTTGCCACTG | Cytokinin-O-glucosyltransferase (CK degradation) | A07_TRINITY_DN52497_c3_g1_i5 |
| Cks | CkZb | GGGGTCTCTGAAAGGCATCC  AGTTCCCCCTACGCTGAGAT | Cytonikin hydroxylase (Zeatin biosynthesis) | C07_S37136 |
| SA | PAD4 | TGATGACCAACCAGCCCTTG  TGAGCCTCTTTTCCAGCACC | Lipase (SA accumulation) | C09_S2708280 |
| SA | ICS | CCTTCTCCAATCTTCCGGCA  GCTAGCGTTGGTGTCTCGAT | isochorismatase synthase family (SA biosynthesis) | A02_TRINITY_DN19059_c0_g2_i3 |
| HG | ACT | GCTAAAGAGCAAGAGACCCC  ATATCGGCACTCCTTCTCAG | Actine | Donoso et al. 2015 |
| HG | UBQ | AGCCCTTATGCCGGAGGGGTTT  AGTGCGGGACTCCACTGTTCCT | Ubiquitine | Sanchez et al. 2003 |

JA: jasmonic acid; SA: salicylic acid; ABA: abscisic acid; GAs: gibberelins; Cks: cytokinins; HG: housekeeping gene.
