# Supplementary material for: A delayed response in phytohormone signaling and production contributes to pine susceptibility to Fusarium circinatum
Source: BMC Plant Biol. 2024 Jul 30;24:727. doi: 10.1186/s12870-024-05342-8 (PMC11289988; doi:10.1186/s12870-024-05342-8)

Supplementary Figure 1: Symptom development in *Pinus pinaster* and *Pinus radiata* at 5, 10, and 21 days post inoculation (dpi) with *Fusarium circinatum*.


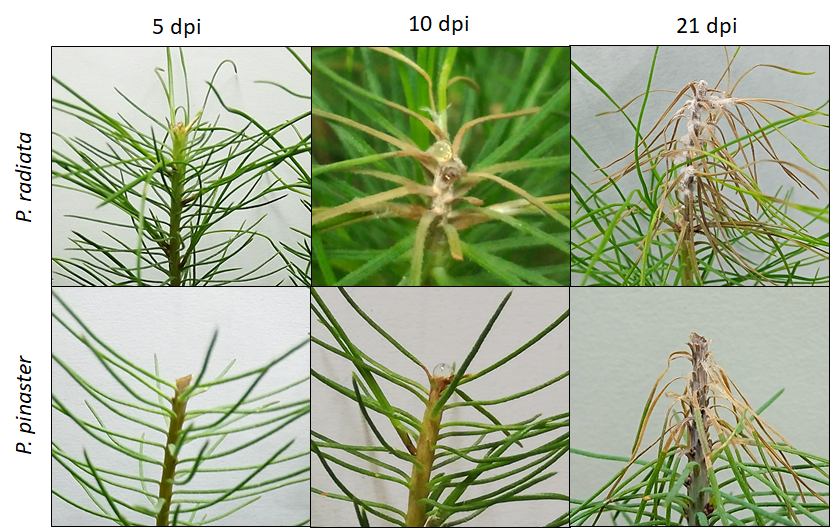

Supplement: Supplementary file 3 — Supplementary Material 3 [file 12870_2024_5342_MOESM3_ESM.docx]
